# The complete genome of a copper-tolerant strain of *Rhodanobacter* from a Superfund site

**DOI:** 10.1128/mra.00403-26

**Published:** 2026-05-18

**Authors:** Mohammed M. A. Ahmed, Paul D. Boudreau

**Affiliations:** 1 Department of Biomolecular Sciences, School of Pharmacy, University of Mississippi 551786, University, Mississippi, USA; 2 Department of Pharmacognosy, Al-Azhar University, Cairo, Egypt; DOE Joint Genome Institute, Berkeley, California, USA

**Keywords:** *Rhodanobacter*, copper, Superfund

## Abstract

A copper-tolerant, siderophore-producing bacterium, *Rhodanobacter* sp. BL-MT-08, was isolated from soil from the Carpenter Snow Creek Mining District Superfund Site in Montana, which has a legacy of heavy metal contamination. The *Rhodanobacter* have been investigated for heavy metal bioremediation, so BL-MT-08 was selected for whole-genome sequencing.

## ANNOUNCEMENT

*Rhodanobacter* have been found to dominate some heavy metal-contaminated environments, with prior genomic studies investigating their systems for heavy metal transport/resistance (1–3). After encountering a copper-tolerant *Rhodanobacter* strain in soil from a heavy metal-contaminated Superfund site, this strain was selected for genome sequencing (4). The original soil sample was collected from the Haystack adit (46.971,483 N 110.719,064 W) on 25 May 2022. To briefly summarize the prior isolation (4), BL-MT-08 was isolated on 2.5 mM copper-treated agar plates of ×1/5 diluted lysogeny broth supplemented with D-amino acids, as described by Nguyen and colleagues (5), as well as trace metals, using the BG-11 trace metal mixture (6) abbreviated as 1/5 LB*. Colonies were restruck onto chrome azurol S dyed plates to identify siderophore-positive colonies, yielding BL-MT-08 as circular yellowish-white colored colonies. A frozen 25% (v/v) glycerol stock of a liquid culture was stored at −70°C. The prior preliminary partial 16S gene sequence was 1,209 bp (4).

**Fig 1:**
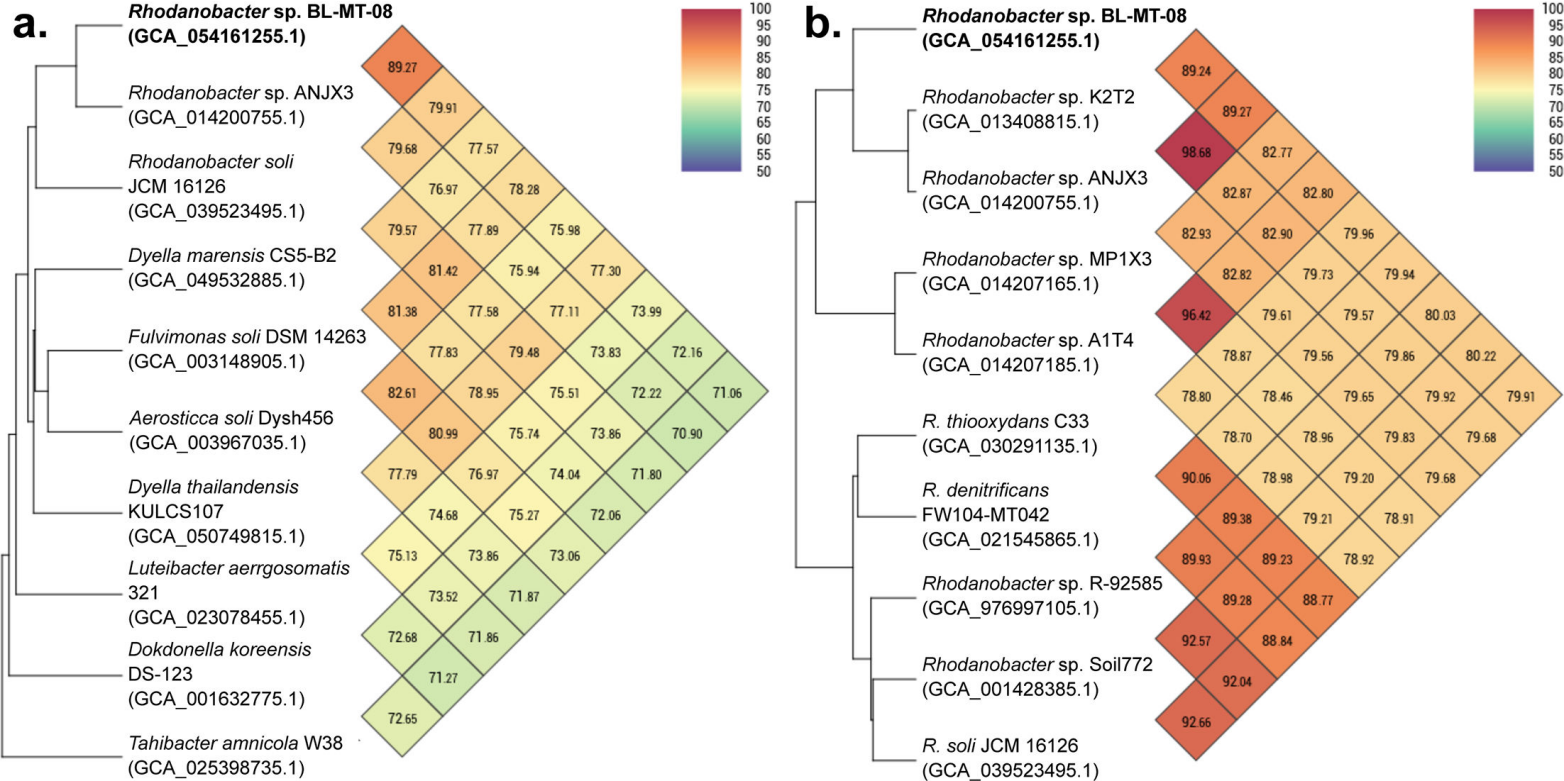
Sequence analysis of the *Rhodanobacter* sp. BL-MT-08 genome. The OrthoANI heatmaps were created with version 0.93.1 of the tool with four threads calculating the orthoANI in both directions with averaging of the result using a genome-to-genome-distance-calculator form of 2 (13). Initially, comparisons of many Rhodanobacteraceae strains were broadly made for the final figure, and a manual selection of strains was used comparing against: (**a**) representative strains across the Rhodanobacteraceae and (**b**) top hits found from the genus *Rhodanobacter*.

Omega BioTek’s E.Z.N.A. Bacterial Kit was used for cell lysis of a 5 mL liquid 1/5 LB* culture started from single-colony frozen stock and grown at 28°C for 3 days. Following the manufacturer’s protocol, TE buffer (100 μL) and lysozyme (10 μL, 50 mg/mL) were added to the cell pellet, and this mixture was incubated at 37°C for 10 min. Then, TL buffer (100 μL) and proteinase K (20 μL, 20 mg/mL) were added for an hour-long incubation at 65°C. Subsequently, 5 μL of RNase (10 mg/mL) was added, and the mixture stood at room temperature for 5 min. After the lysis, the NucleoBond High Molecular Weight (HMW) Kit (Macherey-Nagel) was used to recover HMW DNA following the manufacturer’s instructions. Nanopore DNA sequencing (PromethION with the latest primer-free v14 library prep chemistry) and basecalling (Dorado v4.3, Super-Accurate, with default Q10 quality filtering) by a commercial vendor (Plasmidsaurus, Louisville, Kentucky, USA; Standard Bacteria Genome service) gave 205,010 raw reads with an N50 of 10,419 bp. Reads were filtered with Filtlong (version 0.2.1) first to a minimum length of 500 bp keeping the top 98% of reads by quality score and separately filtering at 1,000 bp/90% (7). The 500 bp/98% filtered reads were assembled via Flye (version 2.9-b1778) with the Nanopore high-quality setting, resulting in one circular contig (252× mean coverage) (8). This draft was polished with Racon (version 1.5.0, default settings) using a minimap2-derived (version 2.17-r941) .sam file generated with the map-ont setting and the 1,000 bp/90% reads. This genome was polished again using the same reads with medaka (version 1.7.2, r941_min_sup_g507 configuration, batch size of 50) (9, 10). The final assembly was 4,233,771 bp with 62.9% guanine + cytosine (G + C) content. CheckM2 (version 1.1.0, default settings) scored the genome as 99.99% complete with 0.07% contamination (11). An Ortho Average Nucleotide Identity (orthoANI) Tool-based (version 0.93.1) comparison to other Rhodanobacteraceae showed orthoANI scores below 90% (Fig. 1a), but with top hits to *Rhodanobacter* spp. (Fig. 1b). A preliminary DFAST annotation (version 1.3.9, default settings) showed this genome had an *FeoAB* operon (3), but antiSMASH (version 8.0.4, relaxed strictness) did not identify any known metallophore pathways (12).

## Data Availability

The final version of the genome assembly for *Rhodanobacter* sp. BL-MT-08, as well as the Sequence Read Archive of the raw vendor reads and preliminary 16S partial gene sequence, are available on GenBank under BioProject number PRJNA1140264. The accession numbers for these data are CP167872.1 for the genome, SRR37849000 for the sequence read archive, PP868359 for the 16S sequence, and SAMN43036439 for the BioSample information on the collection site.
